# Correlation between the Ki-67 proliferation index and response to radiation therapy in small cell lung cancer

**DOI:** 10.1186/s13014-016-0744-1

**Published:** 2017-01-13

**Authors:** Naoya Ishibashi, Toshiya Maebayashi, Takuya Aizawa, Masakuni Sakaguchi, Haruna Nishimaki, Shinobu Masuda

**Affiliations:** 1 Department of Radiology, Nihon University School of Medicine, 30-1 Oyaguchi Kami-cho, Itabashi-ku, Tokyo, 173-8610 Japan; 2 Department of Pathology, Nihon University School of Medicine, Itabashi-ku, Tokyo, 173-8610 Japan

**Keywords:** Ki-67, Proliferation index, Small cell lung cancer, Response, Radiation therapy

## Abstract

**Background:**

In the breast cancer, the decision whether to administer adjuvant therapy is increasingly influenced by the Ki-67 proliferation index. In the present retrospective study, we investigated if this index could predict the therapeutic response to radiation therapy in small cell lung cancer (SCLC).

**Methods:**

Data from 19 SCLC patients who received thoracic radiation therapy were included. Clinical staging was assessed using the TNM classification system (UICC, 2009; cstage IIA/IIB/IIIA/IIIB = 3/1/7/8). Ki-67 was detected using immunostained tumour sections and the Ki-67 proliferation index was determined using e-Count software. Radiation therapy was administered at total doses of 45–60 Gy. A total of 16 of the 19 patients received chemotherapy.

**Results:**

Patients were divided into two groups, one with a Ki-67 proliferation index ≥79.77% (group 1, 8 cases) and the other with a Ki-67 proliferation index <79.77% (group 2, 11 cases). Following radiation therapy, a complete response (CR) was observed in six cases from group 1 (75.0%) and three cases from group 2 (27.3%). The Ki-67 proliferation index was significantly correlated with the CR rate (P = 0.05), which was significantly higher in group 1 than in group 2 (P = 0.04). The median survival time was 516 days for all patients, and the survival rates did not differ significantly between groups 1 and 2.

**Conclusions:**

Our study is the first to evaluate the correlation between the Ki-67 proliferation index and SCLC tumour response to radiation therapy. Our findings suggest that a high Ki-67 proliferation index might represent a predictive factor for increased tumour radiosensitivity.

## Introduction

Ki-67 is a nuclear protein associated with cell proliferation and is expressed in the G1, S, G2 and M phases of the cell cycle but not in the G0 phase [1]. Thus, this protein is used as a marker for the proliferation of various tumour cells. Particularly in breast cancer, Ki-67 positivity is a marker for a high risk of recurrence and poor survival [2], and immunostaining with Ki-67 antibody is routinely used as a proliferation index. In the treatment of breast cancer, Ki-67 is regarded as a predictive marker for the efficacy of chemotherapy, and the decision to administer adjuvant chemotherapy is frequently determined on the basis of the Ki-67 proliferation index [3].

In lung cancer, several studies have reported that high Ki-67 expression was an indicator of poor prognosis in patients with non-small cell lung cancer (NSCLC) [4, 5]. However, few reports have evaluated Ki-67 expression in patients with small cell lung cancer (SCLC). Moreover, the most recent World Health Organization (WHO) classification has adopted the Ki-67 proliferation index for the diagnosis of SCLC, with numerical values of cell proliferation used to diagnose this disease [6]. In the present study, we investigated the association between the Ki-67 proliferation index and the therapeutic effects of radiation therapy in SCLC.

## Patients and methods

Table 1 lists the patient characteristics and the treatment regimens administered.

**Table 1:** Patient characteristics

Patient no.	Sex	Age (yrs)	cstage	Ki-67 (%)	Radiation therapy		Chemotherapy	
					Fraction dose	Total dose	Schedule	Regimen
1	M	69	IIIB	79.77	1.5Gy/fr bid	45Gy	Concurrent	Etoposide + carboplatin
2	M	70	IIA	92.13	1.5Gy/fr bid	45Gy	Neoadjuvant	Etoposide + cisplatin
3	M	80	IIIB	75.87	1.5Gy/fr bid	45Gy	Neoadjuvant	Etoposide + carboplatin
4	F	69	IIA	66.41	2Gy/fr	50Gy	None	
5	M	68	IIIB	45.55	2Gy/fr	50Gy	Neoadjuvant	Etoposide + cisplatin
6	M	84	IIIB	74.84	2Gy/fr	60Gy	None	
7	M	72	IIIA	92.51	2Gy/fr	60Gy	Neoadjuvant	Etoposide + cisplatin
8	M	59	IIIA	74.81	1.5Gy/fr bid	45Gy	Concurrent	Etoposide + cisplatin
9	M	67	IIIA	99.21	1.5Gy/fr bid	45Gy	Concurrent	Etoposide + carboplatin
10	M	66	IIIA	95.74	1.5Gy/fr bid	45Gy	Concurrent	Etoposide + cisplatin
11	M	59	IIIB	89.2	1.5Gy/fr bid	45Gy	Concurrent	Etoposide + cisplatin
12	M	70	IIA	75.34	1.5Gy/fr bid	45Gy	Concurrent	Etoposide + cisplatin
13	M	81	IIB	72.79	3Gy/fr	45Gy	None	
14	F	74	IIIB	92.31	1.8Gy/fr	50.4Gy	Concurrent	Etoposide + cisplatin
15	F	78	IIIB	64.57	2Gy/fr	50Gy	Neoadjuvant	Etoposide + cisplatin
16	M	68	IIIB	78.02	2Gy/fr	50Gy	Neoadjuvant	Etoposide + cisplatin
17	M	69	IIIA	78.57	1.5Gy/fr bid	45Gy	Concurrent	Etoposide + cisplatin
18	M	73	IIIA	98.2	1.5Gy/fr bid	45Gy	Concurrent	Etoposide + cisplatin
19	F	74	IIIA	69.71	1.5Gy/fr bid	45Gy	Neoadjuvant	Etoposide + cisplatin

*Abbreviations*: *M* male, *F* female, *bid* twice daily, *fr* fraction

### Patients

This retrospective study included data from 19 patients (15 males and 4 females) who were diagnosed as having SCLC and received thoracic radiation therapy at our hospital between February 2011 and August 2015.

The patient median age was 70 (range, 59–84) years. Clinical staging was assessed according to the TNM classification (UICC, 2009; cstage IIA/IIB/IIIA/IIIB = 3/1/7/8).

### Ki-67 proliferation index

**Fig. 1:**
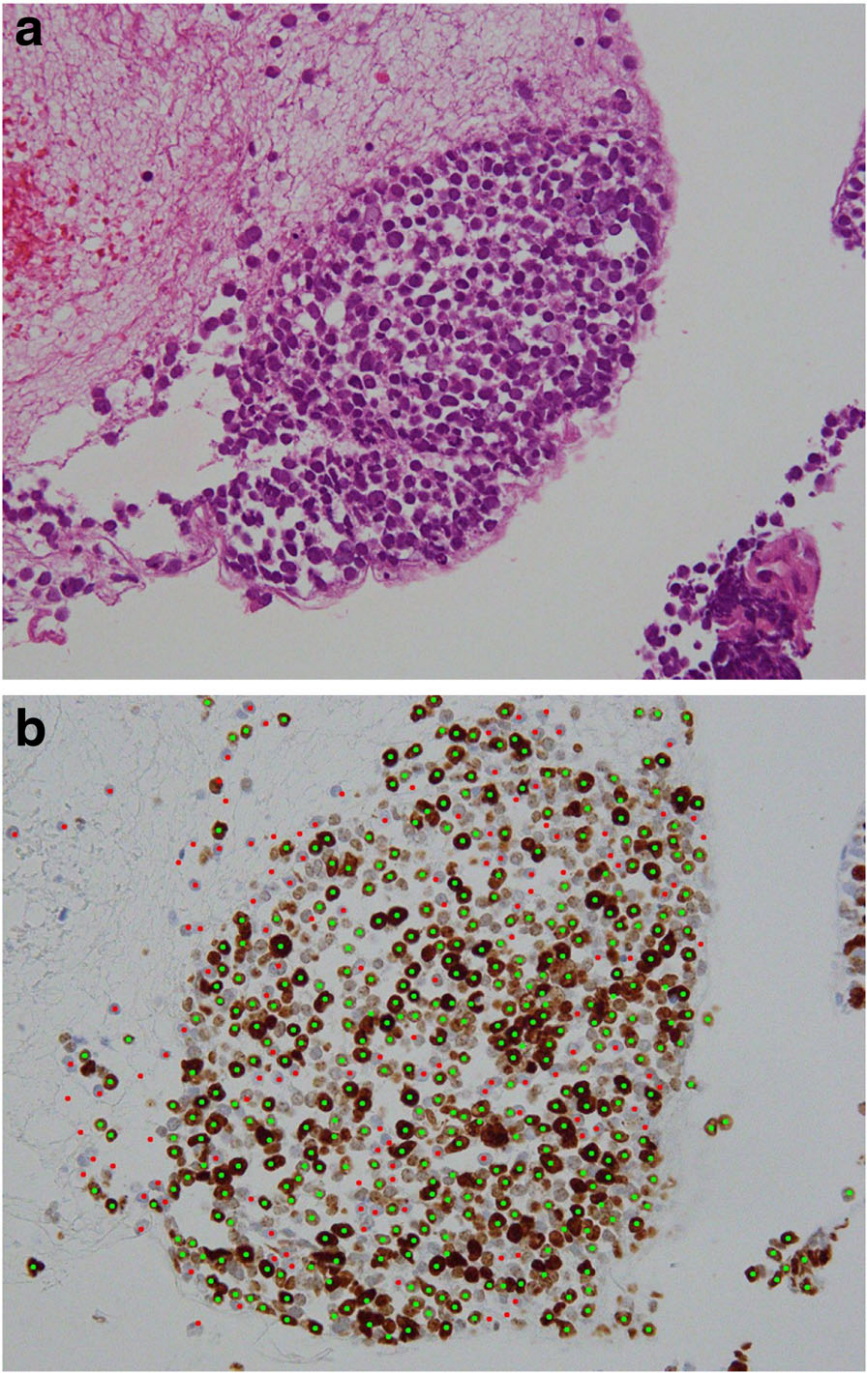
Histological examination of biopsy specimens from lung tumours. **a** Photomicrograph of haematoxylin and eosin-stained tumour section showing small cells with scant cytoplasm and granular nuclear chromatin (objective lens magnification, ×40). **b** Photomicrograph of tumour section immunostained for Ki-67 using the MIB-1 antibody clone, showing positive (green dots) or negative (red dots) cells based on pixel colour intensity. Some cells weakly stained brown were automatically assessed as not positive by the cell counting software

SCLC tumour samples were collected prior to chemotherapy or radiation therapy. Among the 19 patients, bronchoscopic biopsy specimens were collected from 16 patients and percutaneous lung biopsy specimens were collected from 3 patients. Samples were stained with haematoxylin and eosin and the Ki-67 antibody MIB-1 clone (DAKO, Glostrup, Denmark) was used to detect Ki-67 expression. The Ki-67 proliferation index was defined as the percentage of cells with positive nuclear Ki-67 immunostaining in a section of confirmed carcinoma using e-Count cell counting software (e-Path, Kanagawa, Japan). Images of tumour sections mounted on glass slides were converted to JPEG (Joint Photographic Experts Group) format, and cells with positive nuclear immunostaining for Ki-67 were counted based on pixel colour intensity. Images were automatically segmented into Ki-67-positive and Ki-67-negative areas according to the pixel colour intensity cut-off point (Fig. 1).

The cell counting software automatically determines the cut-off point from a histogram of brown density (MIB-1 clone, visualized with DAB labelling) and blue density (haematoxylin) in the nucleus. Tumour samples were also microscopically reviewed by two pathologists to verify the Ki-67-positive and Ki-67-negative scores obtained by the software. The median number of tumour cells was 420 (range, 91–1001) in each sample.

### Thoracic radiation therapy

A linear accelerator was used for 10 MV X-ray irradiation, and some lesions were irradiated with 4 MV X-rays. In principle, multiportal irradiation was applied to the anterior-posterior opposed fields to include at least the primary tumour and metastatic lymph nodes, while regional lymph nodes were included if necessary. All patients who received concurrent chemotherapy were irradiated at 1.5 Gy per fraction twice daily, to a total dose of 45 Gy. Patients irradiated with 2 Gy per fraction to a total dose of 50 or 60 Gy were administered chemotherapy as a neoadjuvant therapy or received radiation therapy alone. In the single patient irradiated with 3 Gy per fraction to a total dose of 45 Gy, the primary tumour was accompanied by an additional non-contiguous tumour nodule in the same pulmonary lobe, and both the tumour and the nodule were irradiated.

### Chemotherapy

A total of 16 of the 19 patients received chemotherapy, which was administered as neoadjuvant therapy in seven patients and as concurrent therapy in nine patients. While 13 patients received a regimen consisting of etoposide and cisplatin, three patients with renal dysfunction received a regimen consisting of etoposide and carboplatin. Three patients who received radiation therapy alone were at an advanced age or had dementia.

### Response to radiation therapy

Tumour responses were assessed using computed tomography (CT), performed after the last day of radiation therapy or chemotherapy (median, 27 [range, 4 − 225] days). Clinical responses were categorized as complete or partial according to the Response Evaluation Criteria in Solid Tumours (RECIST), version 1.1. A complete response (CR) was defined as the disappearance of both the primary tumour and metastatic lymph nodes.

### Statistical methods

SPSS version 21.0 (IBM, Armonk NY, USA) was used for statistical analysis. The therapeutic effects of radiation therapy were analysed using stepwise logistic regression with the following variables: Ki-67 proliferation index (≥mean vs. <mean); age (<median vs. ≥median 70 years); period from the first day of chemotherapy (the first day of radiation therapy in patients who received radiation therapy alone) to the last day of radiation therapy (<median vs. ≥median 39 days); frequency of radiation doses (twice daily vs. once daily); and clinical staging (<IIIB vs. IIIB). The frequency tables for the therapeutic effects of radiation therapy and the Ki-67 proliferation index were analysed using the *χ* ^2^ test. The Kaplan-Meier method was used to estimate the probability of overall survival on the first day of chemotherapy or radiation therapy, whichever came first. Mantel’s log-rank test was performed to compare the differences in survival between the subgroups of patients according to the indicated variables.

## Results

### Ki-67 proliferation index

**Fig. 2:**
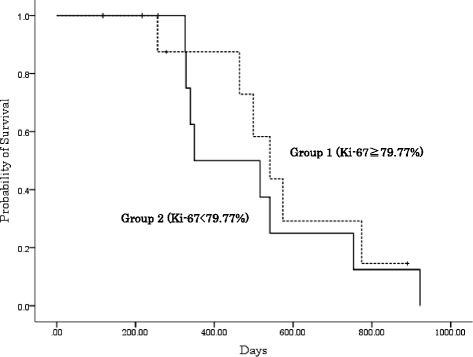
Overall survival curves plotted using the Kaplan-Meier method for patients assigned to the two groups. There was no significant difference observed between the survival rate in groups 1 and 2

The Ki-67 proliferation index ranged from 45.55%–99.21%, with a mean value of 79.77% (Table 1). Patients were classified into two groups, one with a Ki-67 proliferation index ≥79.77% (group 1, 8 cases) and the other with a Ki-67 proliferation index <79.77% (group 2, 11 cases). Following radiation therapy, a CR was observed in six cases from group 1 (75.0%) and three cases from group 2 (27.3%) (Table 2). Stepwise logistic regression analysis revealed that the Ki-67 proliferation index was significantly correlated only with the CR rate (P = 0.05). The *χ* ^2^ test showed that the CR rate was significantly higher in group 1 than in group 2 (P = 0.04). The median survival was 516 days for all patients, and the survival rates in groups 1 and 2 did not differ significantly (Fig. 2). Significant differences were not observed in the other variables.

**Table 2:** Ki-67 proliferation index and patient response to radiation therapy

Group 1 (Ki-67 ≥ 79.77%)			Group 2 (Ki-67 < 79.77%)		
Patient no.	Ki-67	Response	Patient no.	Ki-67	Response
1	79.77	CR	3	75.87	CR
2	92.13	CR	4	66.41	CR
7	92.51	CR	5	45.55	PR
9	99.21	CR	6	74.84	PR
10	95.74	PR	8	74.81	CR
11	89.2	PR	12	75.34	PR
14	92.31	CR	13	72.79	PR
18	98.2	CR	15	64.57	PR
			16	78.02	PR
			17	78.57	PR
			19	69.71	PR
		CR rate 75.0% (*p* = 0.04^a^)			CR rate 27.3%

*Abbreviations*: *CR* complete response, *PR* partial response

^a^χ^2^ test

## Discussion

The WHO Classification of Tumours of the Lung, Pleura, Thymus and Heart (fourth edition; 2015) was the first classification system to adopt the Ki-67 proliferation index for the differentiation of neuroendocrine tumours. This classification indicates that, based on previous studies involving biopsy samples or surgical specimens [7, 8], the Ki-67 proliferation index in SCLC is typically >50%, ranging from 50% to 100%; in addition, cell proliferation is prominent in SCLC [6]. However, in the diagnosis of SCLC, the differentiation of this tumour from carcinoid tumours remains a challenge. Because the standard treatment for SCLC is chemoradiotherapy, large tumour samples are rarely obtained during surgery and the diagnosis is instead routinely made based on small tumour samples obtained using bronchoscopy or other procedures. As a result of limitations such as the presence of crush artefacts and poor tissue preservation, the use of cytoplasmic markers may lead to inaccurate diagnoses. The nuclear marker Ki-67, which is well preserved in samples with an extensive crush artefact, can effectively differentiate SCLC from carcinoid tumours [7, 8]. A further challenge associated with the diagnosis of lung tumours is the potential tumour heterogeneity observed between biopsy samples and surgical specimens. However, in NSCLC, high correlation has been reported between the expression of Ki-67 in biopsy samples and surgical specimens [9]. An additional limitation associated with the Ki-67 proliferation index is the lack of consistent counting methods, which has caused variations among pathologists in the index calculated [10]. Thus, to reduce these variations as much as possible in the present study, we used e-Count, a cell counting software program that counts cells with a nucleus that is positive for Ki-67 immunostaining on the basis of pixel colour intensity. Our study is the first to use e-Count (e-Path, Kanagawa, Japan). A recent study has reported the use of digital image analysis in Ki-67 immunostaining; in this study, digital image processing software was used to reduce variations in the Ki-67 proliferation index [11].

Regarding the association between the Ki-67 proliferation index and lung cancer, many studies on NSCLC (including meta-analyses) have indicated that Ki-67 expression is a poor prognostic factor for survival [4, 5]. In contrast, only two studies to date have evaluated the association between SCLC and the Ki-67 proliferation index, one of which reported that the outcome was poor in patients with biopsy samples yielding a Ki-67 proliferation index lower than the median [12]. Another study has reported that there was no association between survival and the Ki-67 proliferation index in SCLC [13]. Thus, the significance of the Ki-67 proliferation index in SCLC has been controversial. In NSCLC, the excision repair cross-complementation group 1 (ERCC1) protein is reported to be associated with resistance to platinum-based chemotherapy. In SCLC, however, ERCC1 was not related to survival or to chemoradiation therapy response, and no association was observed between Ki-67 and ERCC1 [13]. In vitro, SCLC cell lines are sensitive to radiation, and the dose-response curves for these cells lack a shoulder. Furthermore, relatively low radiation doses per fraction have been shown to be lethal to SCLC cell lines [14]. On the basis of these radiobiological features, it is known that twice-daily thoracic radiation therapy improves overall survival when this therapy is initiated with the first cycle of chemotherapy or less than 30 days after the start of the first cycle of chemotherapy [15, 16]. Moreover, according to the “law of Bergonié and Tribondeau” (1906), which established a link between cell proliferation and cellular radiosensitivity, cells that more frequently undergo cell division are more sensitive to radiation. Proliferating cells have been reported to be more sensitive to radiation than quiescent cells in vitro [17]. Ki-67 was originally obtained from monoclonal antibodies to the nuclear antigen in Hodgkin and Sternberg-Reed cells. The nuclear antigen detected by Ki-67 is expressed in almost all human cell lines, but is not expressed in normal human cells in the resting stage. Ki-67 thus recognizes a nuclear antigen associated with cell proliferation [1].

Thus, we expected that tumour cells would have a higher rate of proliferation, more frequently undergo cell division, and be more sensitive to radiation in patients with a higher Ki-67 proliferation index before treatment; when we compared the mean Ki-67 proliferation index of the two groups (≥79.77% vs. <79.77%), tumour responses in terms of the CR rate were greater in the group with a Ki-67 proliferation index equal to or higher than the mean, as we expected. Few previous reports have evaluated the association between the Ki-67 proliferation index and tumour responses to radiation therapy. A study on uterine cervical cancer demonstrated that patients with a higher Ki-67 proliferation index at the time of diagnosis showed a significantly better histological response to radiation therapy at a total dose of 30 Gy [18]. Another study on oral squamous cell carcinoma (OSCC) reported that the Ki-67 proliferation index at the time of diagnosis had no significant correlation with the response to radiation therapy; in contrast, the reduction in the growth fraction (decrease in proliferation index) after radiation therapy at a total dose of 10 Gy was significantly correlated with the CR rate [19]. A study of OSCC after curative resection and postoperative radiation therapy reported that low Ki-67 proliferation index tumours had a significantly shorter time to recurrence than high proliferation index tumours [20]. This study concluded that tumours with a high Ki-67 proliferation index might respond better to radiation therapy as a result of increased radiosensitivity. Two studies on rectal cancer have reported results that were contradictory: one showed that there was no correlation between the Ki-67 proliferation index and the rate of response to radiation therapy, while the other reported that there was a good correlation between high Ki-67 proliferation index and improved response to radiation therapy [21, 22]. Our study is the first to evaluate the correlation between the Ki-67 proliferation index and the rate of response to radiation therapy in SCLC. Our findings suggest that a higher Ki-67 proliferation index might represent a predictive factor for higher radiosensitivity. Although we also examined whether the Ki-67 proliferation index was a prognostic factor in SCLC, as observed for NSCLC in previous studies, no difference in the survival rate was observed between groups 1 and 2. This may have been attributable to the fact that all three patients who were unable to receive chemotherapy were included in group 2 and had a Ki-67 proliferation index that was lower than the mean; as mentioned, a higher Ki-67 proliferation index might be associated with a higher survival rate.

A challenge for future studies is the low number of tumour cells in each sample. In the present study, the mean number of tumour cells was 420, which is lower than the number required (≥500 cells) for the assessment of Ki-67 in breast cancer, for example, according to the Ki-67 proliferation index guidelines [23]. Given that large SCLC samples are rarely obtained from surgery, there is a need to develop a technique that consistently obtains an adequate number of tumour cells, even from small samples acquired using bronchoscopy or other procedures. Further studies among SCLC patients are needed to assess the significance of the Ki-67 proliferation index in the treatment of SCLC.

## Conclusions

To the best of our knowledge, our study is the first to evaluate the correlation between Ki-67 proliferation index and SCLC tumour response to radiation therapy. Our findings suggest that a higher Ki-67 proliferation index might represent a predictive factor for increased tumour radiosensitivity.

## Abbreviations

CR: Complete response
CT: Computed tomography
NSCLC: Non-small cell lung cancer
OSCC: Oral squamous cell carcinoma
RECIST: Response evaluation criteria in solid tumours
SCLC: Small cell lung cancer
WHO: World Health Organization
